# Anti-class a scavenger receptor autoantibodies from systemic lupus erythematosus patients impair phagocytic clearance of apoptotic cells by macrophages *in vitro*

**DOI:** 10.1186/ar3230

**Published:** 2011-01-31

**Authors:** Xiao-wei Chen, Yan Shen, Chuan-yin Sun, Feng-xia Wu, Yi Chen, Cheng-de Yang

**Affiliations:** 1 Department of Rheumatology, Renji Hospital, Shanghai Jiaotong University School of Medicine, 145 Shan Dong Middle Road, Shanghai 200001, PR China; 2 Department of Rheumatology, the first affiliated hospital of Wenzhou Medical College, 2 Fu Xue Alley, Zhejiang 325000, PR China

## Abstract

**Introduction:**

Inadequate clearance of apoptotic cells by macrophages is one of the reasons for the breakdown of self-tolerance. Class A scavenger receptors, macrophage receptor with collagenous structure (MARCO) and scavenger receptor A (SR-A), which are expressed on macrophages, play important roles in the uptake of apoptotic cells. A previous study reported the presence of the anti-MARCO antibody in lupus-prone mice and systemic lupus erythematosus (SLE) patients. The purpose of this study was to investigate the prevalence of anti-class A scavenger receptor antibodies in patients with various autoimmune diseases, in particular SLE, and the functional implication of those autoantibodies in the phagocytic clearance of apoptotic cells by macrophages.

**Methods:**

Purified recombinant scavenger receptor cysteine-rich (SRCR) polypeptide (ligand-binding domain of MARCO) and recombinant SR-A were used as antigens. By using enzyme-linked immunosorbent assay, the anti-SRCR and anti-SR-A antibodies were detected in the sera of untreated patients with SLE (n = 65), rheumatoid arthritis (n = 65), primary Sjögren syndrome (n = 25), and healthy blood donors (n = 85). The effect of IgG purified from SLE patients or healthy controls on the phagocytosis of apoptotic cells by macrophages was measured by the flow cytometry assay.

**Results:**

Anti-SRCR antibodies were present in patients with SLE (18.5%) and rheumatoid arthritis (3.1%), but not in those with primary Sjögren syndrome. Anti-SR-A antibodies were present in patients with SLE (33.8%), rheumatoid arthritis (13.8%), and primary Sjögren syndrome (12.0%). IgG from SLE patients positive for anti-SRCR or anti-SR-A antibodies showed a higher inhibition rate on binding of apoptotic cells to macrophages than IgG from healthy controls (both *P *< 0.05). IgG from SLE patients positive for both anti-SRCR and anti-SR-A antibodies showed a significantly higher inhibition rate on ingestion of apoptotic by macrophages than IgG from healthy controls (*P *< 0.05).

**Conclusions:**

Our results indicated that autoantibodies to class A scavenger receptors might contribute to the breakdown of self-tolerance by impairing the clearance of apoptotic debris and play a role in the pathogenesis of autoimmune disease, especially in SLE.

## Introduction

Systemic lupus erythematosus (SLE) is a systemic autoimmune disease characterized by the production of a wide range of autoantibodies. Several lines of evidence suggest that increased apoptosis and impaired phagocytic clearance of apoptotic cells could play important roles in the breakdown of self-tolerance because they lead to autoantigen overload, a decrease in anti-inflammatory cytokine production, and in susceptible individuals, initiation of an autoimmune response [1]. Studies on human SLE have shown increased apoptosis of peripheral blood mononuclear cells, neutrophils, and macrophages [2,3]. In addition to aberrant apoptosis, macrophages from patients with SLE exhibited impaired clearance of apoptotic cells both *in vitro *and *in vivo *[3-6].

However, it is unclear whether macrophages of patients with SLE have intrinsic defects resulting in reduced clearance of apoptotic cells; serum factors have been implicated in the reduced clearance of apoptotic cells by macrophages. The complex process of phagocytosis involves a range of receptors, ligands, and opsonins, which are involved in the recognition and internalization of apoptotic cells. Recent studies indicated that class A scavenger receptor member macrophage receptor with collagenous structure (MARCO) and scavenger receptor A (SR-A) could bind to apoptotic cells and contribute to the clearance of apoptotic cells [7,8]. Interestingly, subsequent study by Wermeling *et al. *[8] showed that FcγRIIB^-/- ^(NZB × NZW) F1 mice, which develop spontaneous SLE, produce autoantibodies that are capable of recognizing MARCO and SR-A. Study has also shown that patients with SLE also produce autoantibodies to MARCO. It is proposed that autoantibodies blocking scavenger receptors may alter the phagocytosis of apoptotic cells by macrophages, and, thereby, facilitate the development of an autoimmune response.

Class A scavenger receptors appear to be new target antigens in SLE. MARCO is a trimeric membrane protein containing a short N-terminal intracellular domain, a transmembrane domain, and a large extracellular domain composed of a spacer domain, a long collagenous domain, and a C-terminal scavenger receptor cysteine-rich (SRCR) domain. SRCR plays a major role in the ligand-binding function of MARCO [9,10]. The binding of autoantibodies to MARCO in (NZB × NZW) F1 mice could be blocked by an antibody to SRCR [8], implicating SRCR as a potential autoantigen in SLE. SR-A, the other class A scavenger receptor member that is also capable of binding with apoptotic cells, is structurally quite similar to MARCO; however, it differs from MARCO in that it has an a-helical coiled-coil domain, but a short collagenous domain and its ligand-binding function have been localized to the collagenous domain. Considering the possibility that SRCR and SR-A could serve as autoantigens in autoimmune diseases, we evaluated the prevalence of anti-SRCR antibodies and anti-SR-A antibodies in patients with SLE and other various systemic autoimmune diseases. Furthermore, we investigated whether these autoantibodies could interfere with the phagocytic clearance of apoptotic cells by macrophages.

## Materials and methods

### Patients and healthy controls

All patients in this study were followed at the Renji Hospital affiliated with Shanghai Jiaotong University School of Medicine, Shanghai, China. The consecutively recruited patients include 65 untreated new onset patients with SLE who fulfilled the American College of Rheumatology (ACR) classification criteria for the diagnosis of SLE [11], 65 patients with rheumatoid arthritis (RA) who satisfied the ACR criteria for RA [12], 25 patients with primary Sjögren syndrome (pSS) diagnosed according to the American-European consensus group criteria [13], and 85 healthy blood donors. Demographic data were collected for all patients (expect for RA patients) and have been presented in Table 1. All the serum samples were stored at -80°C. Disease activity was measured using the SLE disease activity index 2000 (SLEDAI-2K) [14]. The main clinical and serological features of SLE patients are shown in Additional file 1. The patients were informed of the purpose of the study and provided their consent for the study. The institutional review broad of Shanghai Jiaotong University approved this study.

**Table 1:** Demographic characteristics of patients

	Number of patients	Age (years)	Female/male
Healthy control	85	33 (27 to 44)	74/11
SLE	65	31 (25 to 42)	59/6
pSS	25	42 (31 to 51)	24/1
RA	65	NA	NA

Values for age are expressed as median (25^th ^to 75^th ^percentile). There were no significant differences between patients with SLE and healthy donors in terms of age and sex. NA indicates not available. pSS, primary Sjögren syndrome; RA, rheumatoid arthritis; SLE, systemic lupus erythematosus.

### Production and purification of the recombinant human SRCR polypeptide

The SRCR domain (residues 421 to 520) of the human MARCO protein was expressed in *Escherichia coli *as a six-residue poly-histidine fusion protein by using the pET-28a vector (Novagen, Darmstadt, Germany). The DNA fragment encoding SRCR was generated by polymerase chain reaction and the sequence were confirmed by DNA sequencing. The recombined protein produced in bacteria was purified using Ni-NTA resin (Qiagen, Valencia, CA, USA), and the purity of sample was determined to be >85% by performing sodium dodecyl sulfate-polyacrylamide gel electrophoresis. The SRCR domain of MARCO was identified by matrix-assisted laser desorption/ionisation-time of flight mass spectrometry, as described previously [9]. The recombined protein was stored in small aliquots at -80°C.

### Anti-class A scavenger receptors autoantibodies assays

For the detection of anti-SRCR and anti-SR-A antibodies, high-binding plates (Costar, Cambridge, MA, USA) were coated with 10 μg/ml SRCR or 2 μg/ml human SR-AI (R&D Systems, Inc. Minneapolis, MN, USA) in 0.01 M phosphate-buffered saline (PBS) overnight at 4°C. The plates were washed three times with PBS plus 0.05% Tween 20 and blocked with PBS containing 0.3% bovine serum albumin for two hours at 37°C. The blocking buffer was tapped off and washed as described above before the addition of 100 μl of serum (1:100 diluted in blocking buffer). Serum samples of patients with high titers of anti-SRCR or anti-SR-A in the pilot enzyme-linked immunosorbent assay (ELISA) were used as positive controls. The plates with samples were incubated for one hour at 37°C. After washing the plates in the manner described above, the bound human IgG was detected by horseradish peroxidase-conjugated goat anti-human IgG (Fc specific; Sigma-Aldrich, St. Louis, MO, USA). After an additional incubation for one hour at 37°C, the plates were washed and 100 μl of the tetramethylbenzidine/H_2_O_2 _substrate solution (Kirkegard and Perry Labs, Gaithersburg, MD, USA) was added. The color development was stopped by the addition of 50 μl of 0.5 M H_2_SO. Absorbance was measured at a wavelength of 450 nm in a microplate reader (Bio-Rad Laboratories, Hercules, CA, USA). All samples were run in duplicates and corrected for background binding. For the anti-SRCR or anti-SR-A antibody, the mean optical density (OD) value plus three times the standard deviation (SD) of the values determined for sera collected from healthy controls was used as the cutoff for determining positivity.

### Apoptotic cells

Jurkat cells (a human T-cell line) were cultured in RPMI 1640 (Gibco BRL, Gaithersburg, MD, USA) containing 10% heat-inactivated fetal calf serum (FCS) (Hyclone, Beijing, China), 100 U/ml penicillin, and 100 U/ml streptomycin (Hyclone) at 37°C in a humidified 5% CO_2 _incubator. Before the induction of apoptosis, Jurkat cells were loaded with carboxyfluorescein diacetate succinimidyl ester (CFSE) (Molecular Probes, Eugene, OR, USA) according to the manufacturer's protocol (5 μM per 1 × 10^7^cells, for five minutes at 37°C). Then, the cells were washed with 10% FCS-RPMI. Apoptosis was induced by irradiation with ultraviolet B light, as described previously [6]. Induction of apoptosis in Jurkat cell by this method has previously proven to be highly reproducible, as assessed by flow cytometry by PE-conjugated annexin V and 7-amino-actinomycin D (7-AAD) staining (BD Pharmingen, San Diego, CA, USA).

### IgG purification

IgG were isolated from the sera of patients and healthy donors by using the protein G affinity column (Pierce, Rockford, IL, USA); the isolates were examined for endotoxin contamination by using the Limulus amoebocyte lysate kit (A&C Biological Ltd., Zhangjiang, China). The total protein content was estimated by the BCA kit (Pierce).

### Antigen adsorbed IgG

In order to confirm it is the specific activity between the anti-Class A scavenger receptors antibodies and target antigens that impairs phagocytosis, we carried out the phagocytosis assay by removal of specific antibodies against SRCR and SR-A. High-biding plates were coated with antigen and blocked as described above in the ELISA tests. Purified IgG from each patient (diluted in culturing medium for macrophage) were added to wells coated with antigens to adsorb anti-SR-A and anti-SRCR antibodies, and the same samples were also added to wells coated with PBS as controls. Unbound IgG from antigen-coated and non-antigen-coated wells were collected and incubated with macrophage, respectively. Phagocytosis tests were performed as described below.

### Preparation of monocyte derived macrophages

The human monocytic cell line THP-1 cells (ATCC, Manassas, Va, USA) were cultured in the same medium as that used for culturing the Jurkat cells. THP-1 were plated onto 48-well plates at a concentration of 1 × 10^5 ^cells/well; 100 nM phorbol 12-myristate 13-acetate (PMA) (Sigma-Aldrich, St. Louis, MO, USA) was then added to the wells and maintained for 24 hours to induce THP-1 cell differentiation into macrophage. The next day, PMA and the non-adherent cells were washed off three times with 0.1 M PBS, resulting in adherent macrophages on the plates. THP-1 derived macrophages by this way were confirmed to express SR-A and MARCO [15,16]. The macrophages were then incubated with fresh medium containing IgG that was purified from sera with or without anti-class A scavenger receptor antibody for one hour at 37°C in a humidified 5% CO_2 _incubator.

### Phagocytosis assays

In each phagocytosis assay, cells are cultured in the same plate and culture conditions. After incubation with purified IgG, THP-1-derived macrophages were washed three times with the culturing medium to remove non-binding IgG, and CFSE-labeled apoptotic and nonapoptotic Jurkat cells (5 × 10^5 ^cells per well) were added to each well to incubate with the macrophages for one hour. After incubation, the non-binding Jurkat cells were removed by repeated washing with cold PBS. The remaining firmly adherent cells were trypsinized (0.05% tripsin, Hyclone, Beijing, China), harvested in PBS/1%BSA, and stained with PERCP-conjugated mouse anti-human CD3 mAb (BD Pharmingen, SanDiego, CA, USA). Flow cytometry assay was carried out on an FACSCalibur instrument with CellQuest software (both from BD Bioscience, Mountain View, CA, USA). Macrophages with ingested apoptotic Jurkat cells were CFSE^+^CD3^-^, while macrophages with bound apoptotic Jurkat cells were CFSE^+^CD3^+^. Percentages of positive cells were analyzed by using the CellQuest software. The inhibition effect of IgG on macrophage binding or ingestion of apoptotic cell was defined as inhibition rate, and the percentages of macrophages binding/ingestion of apoptotic cells in wells pretreated with PBS rather than IgG were set as 100%. Inhibition rate (%) = (percentage of binding/ingestion in PBS pretreated well-percentage of binding/ingestion in IgG pretreated well)/percentage of binding/ingestion in PBS pretreated well × 100.

### Statistical analysis

For continuous variables, the comparisons were carried out using the unpaired *t*-test for two independent samples, the Mann-Whitney *U *test for non-normal data and paired t-test for matched samples. Categorical variables between different groups were compared by the chi-square test or Fisher's exact test when required. Correlations between groups were evaluated by the Spearman test. A *P-*value less than 0.05 was considered statistically significant. Data were analyzed using the SPSS software for Windows (Version 11.0; SPSS Inc, Chicago, IL, USA).

## Results

### Prevalence of anti-Class A scavenger receptors antibodies

**Figure 1:**
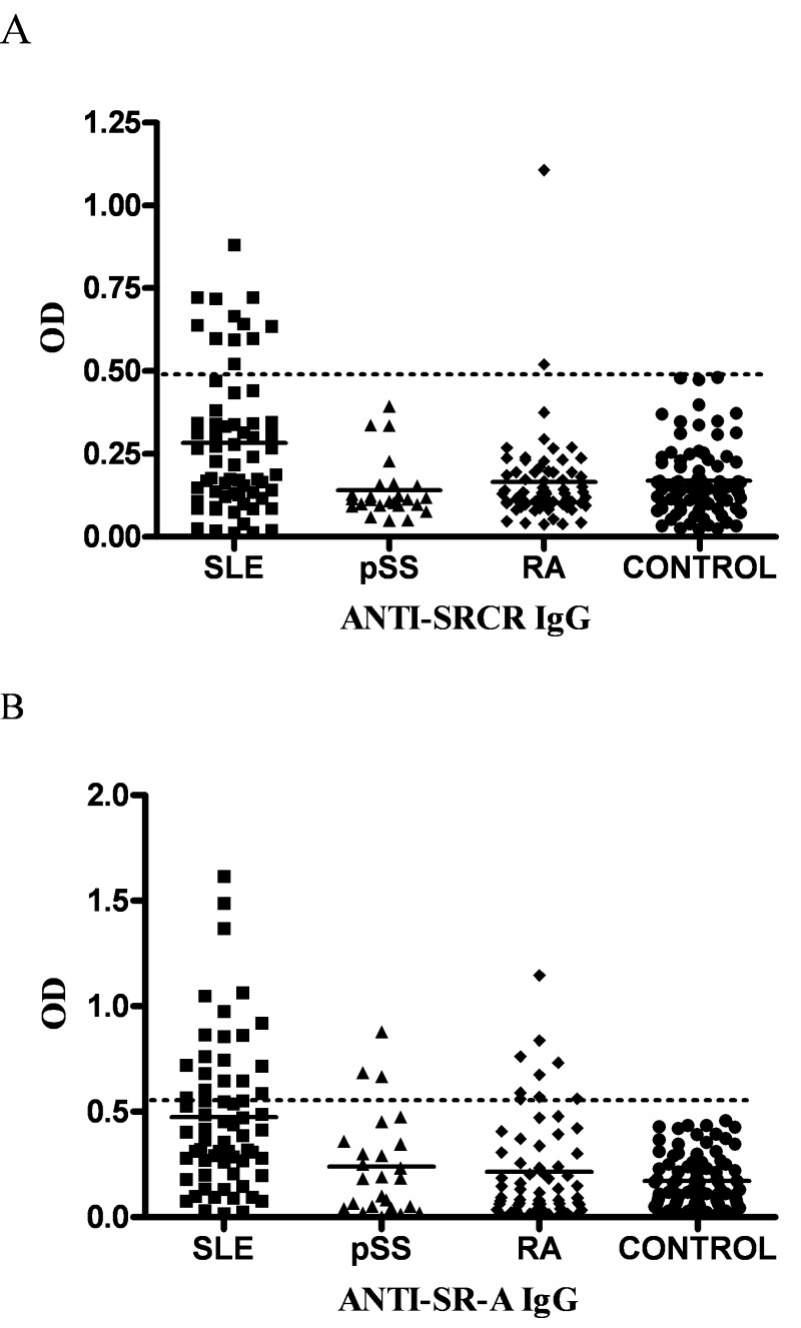
**Levels of anti-class A scavenger receptors IgG in patients and healthy individuals**. The optical density (OD) value for each individual is represented as a single point. Dashed lines indicate the OD values that exceed the mean control value by more than three standard deviations (SDs). Horizontal bars represent the mean values for each group. CONTROL, healthy donors; pSS, primary Sjögren syndrome; RA, rheumatoid arthritis; SLE, systemic lupus erythematosus. **A**. Levels of anti-SRCR IgG for patients. **B**. Levels of anti-SR-A IgG for patients.

For each individual, the OD values for anti-SRCR and anti-SR-A IgG (Figure 1A, B) were plotted. None of the 85 healthy individuals was anti-SRCR or anti-SR-A positive (Table 2). Among the 65 patients with SLE, the prevalence of anti-SRCR and anti-SR-A antibody, as determined by ELISA, was 12 (18.5%) and 22 (33.8%), respectively. The prevalence of anti-SRCR or anti-SR-A did not show any statistically significant association with sex or age (data not show). The frequency of anti-SRCR was higher in patients with SLE than that in patients with RA or pSS (*P *= 0.005 and *P *= 0.032, respectively). A higher frequency of anti-SR-A positivity was observed in SLE patients than in RA or pSS patients (*P *= 0.007 and *P *= 0.038, respectively). There was not a statistically significant correlation between anti-SR-A and anti-SRCR level (*P *> 0.05).

**Table 2:** Prevalence of anti-SRCR and anti-SR-A IgG in patients and healthy controls

	Anti-SRCR positive (%)	Anti-SR-A positive (%)	Double positive (%)
Healthy control (n = 85)	0	0	0
SLE (n = 65)	18.5*	33.8*	7.7^†^
RA (n = 65)	3.1^§^	13.8*	0
pSS (n = 25)	0	12.0*	0

* *P *< 0.001 as compared with healthy controls.

^†^*P *= 0.014 as compared with healthy controls.

^§ ^*P *= 0.186 as compared with healthy controls.

pSS, primary Sjögren syndrome; RA, rheumatoid arthritis; SLE, systemic lupus erythematosus; SR-A, scavenger receptor A; SRCR, scavenger receptor cysteine-rich.

### Inhibition of macrophage uptake of apoptotic cells by anti-class A scavenger receptors autoantibodies

**Figure 2:**
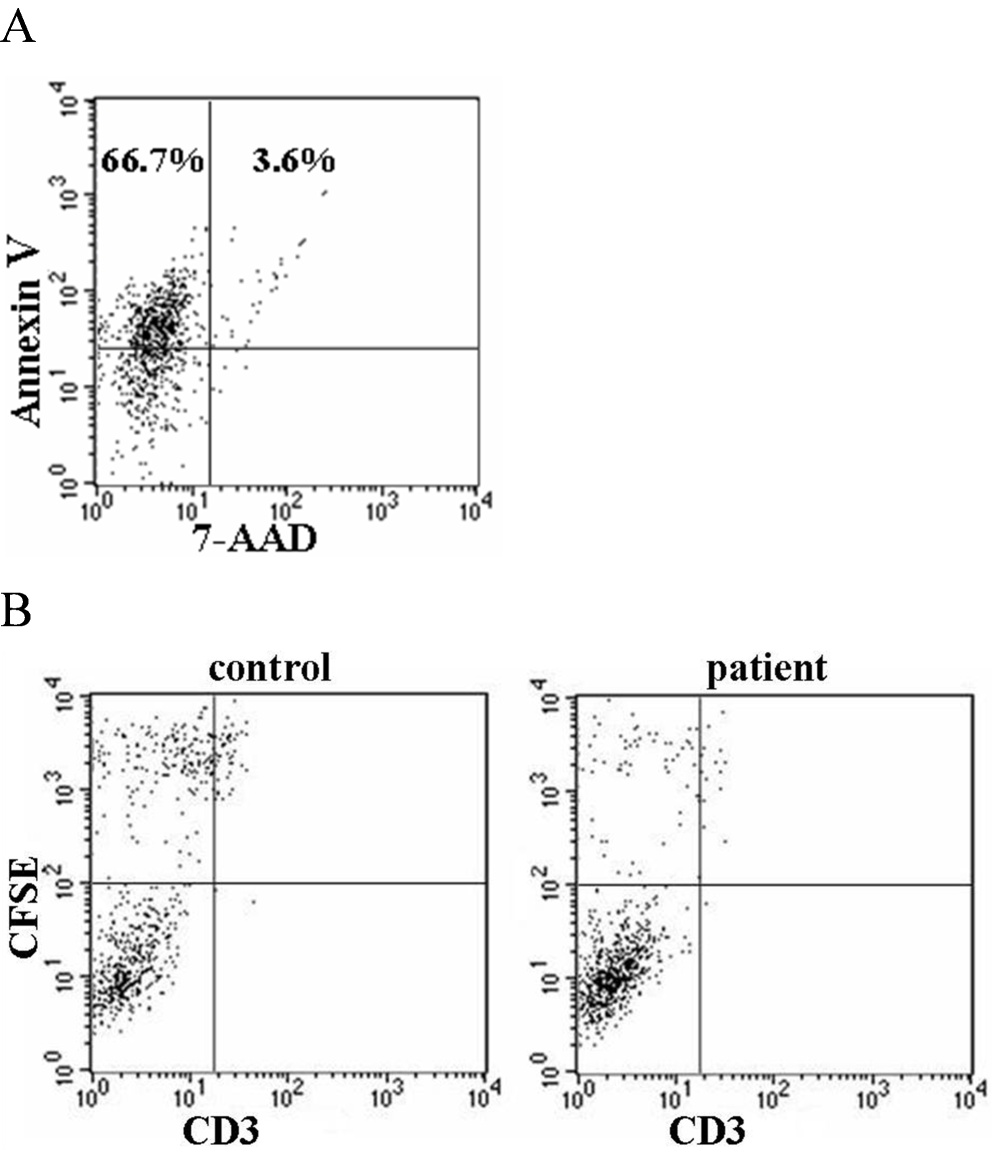
**THP-1 derived macrophage phagocytosis of apoptotic Jurkat cells**. **A**. Apoptosis induction was confirmed by the detection of percentage of Annexin V and 7-AAD staining by flow cytometric assessment. More than 50% cells were Annexin V ^+^7-AAD^-^(early apoptotic cells) and less than 5% cells were Annexin V ^+^7-AAD^+ ^(late apoptotic and necrotic cells) after ultraviolet B irradiation. This analysis was repeated prior to each phagocytosis assay. One representative image is shown. **B**. Flow cytometry based phagocytosis assay. To discriminate between binding and internalization of apoptotic Jurkat cells, CD3 staining of CFSE^+ ^macrophages was performed. Macrophages that had ingested apoptotic Jurkat cells were CD3^- ^(upper left quadrant), while macrophages with Jurkat cells bound to their surface were CD3^+ ^(upper right quadrant). Macrophages were pretreated with IgG from SLE patients or healthy controls before being incubated with apoptotic cells, as described in Materials and Methods. Representative flow cytometry images for phagocytosis by macrophages pretreated with IgG from control (left panel) and SLE patients with anti-class A scavenger receptor antibodies (right panel) are shown.

**Figure 3:**
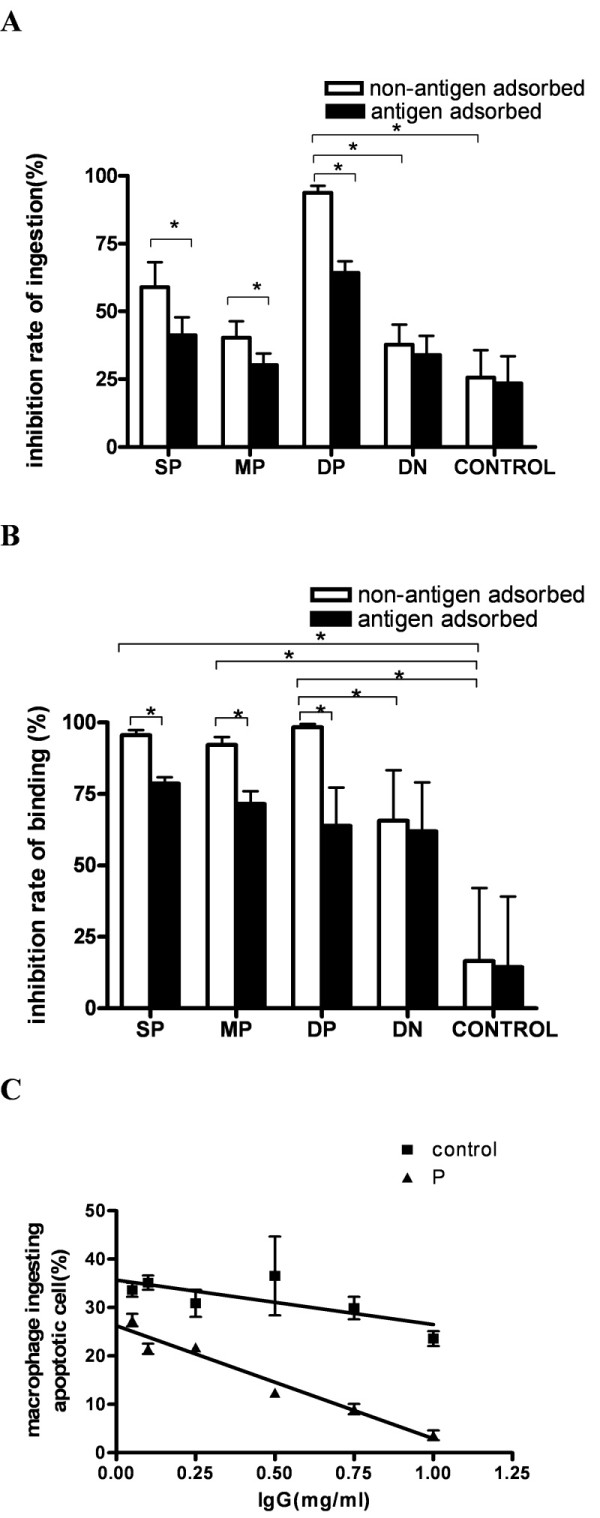
**Phagocytosis of apoptotic cells by macrophages that were pretreated with purified IgG**. **A**, **B**. Macrophages were pre-treated with IgG from SLE patients who were anti-SR-A positive (SP), anti-SRCR (anti-MARCO) positive (MP), anti-SR-A and anti-SRCR double-positive (DP), anti-SRCR and anti-SR-A double negative (DN), or IgG from healthy controls who are anti-SR-A and anti-SRCR double negative (CONTROL) before being cultured with apoptotic cells. Purified IgG were incubated with SRCR and SRA (antigen adsorbed) or not (non-antigen adsorbed) before added to interact with macrophage as described in Materials and Methods. Pre-incubation with DP reduced the percentage of macrophages ingesting apoptotic cells, resulted in a significantly higher inhibition rate of ingestion. Pre-incubation with SP, MP, and DP reduced the percentage of macrophages with apoptotic cells binding to their surface, as inhibition rates significantly increased. The inhibition rates significantly decreased when anti-SRCR and anti-SRA antibodies had been adsorbed off (all *P *< 0.05). An * indicates a statistically significant difference *P *< 0.05 by Mann-Whitney *U *test or paired *t-*test. Each bar represents the mean + SEM (*n *= 4). Results are representative of three experiments. **C**. Dose response of the IgG mediated inhibition of phagocytosis. Decreased percentage of macrophages ingesting apoptotic cells correlated with increased concentration of incubating IgG from one SLE patient positive for both anti-SRCR and anti-SR-A (P) (*r *= -0.943, *P *= 0.005). Concentration of IgG from one healthy control (control) did not correlate with the percentage of macrophages ingesting apoptotic cells (*P *= 0.208). Bar represents the mean ± SEM (*n *= 2). Results are representative of three experiments.

THP-1-derived macrophages were used to assess the uptake of apoptotic Jurkat cells in the absence of human serum. Apoptosis of Jurkat cells was confirmed by annexin V and 7-AAD staining (Figure 2A). CD3 staining was analyzed to discriminate between the binding and ingestion of apoptotic Jurkat cells (Figure 2B). To test the inhibitory effect of anti-class A scavenger receptor autoantibodies, we isolated IgG from four SLE patients with anti-SRCR (+) anti-SR-A (+), four SLE patients with anti-SRCR (+) anti-SR-A(-), four SLE patients with anti-SRCR (-) anti-SR-A (+), four SLE patients with anti-SRCR (-) anti-SR-A (-) and four healthy controls. The inhibition rates of phagocytosis for each sample were calculated. IgG with both anti-SRCR and anti-SR-A antibodies showed higher inhibition rate of ingestion of apoptotic cells by macrophages than IgG from healthy controls (*P *< 0.050) (Figure 3A). IgG positive for anti-SRCR or anti-SR-A antibodies showed higher inhibition rates of ingestion of apoptotic cells by macrophages compared to IgG from normal controls; however, these differences were not statistically significant (both *P *> 0.05). The inhibition rates of binding for IgG samples with anti-SR-A or anti-SRCR antibodies were higher compared to IgG from healthy control (both *P *< 0.050) (Figure 3B). The inhibition effects of IgG with anti-class A scavenger receptor antibodies (non-antigen adsorbed) on binding/ingestion of apoptotic cells by macrophage decreased when anti-class A scavenger receptor antibodies been adsorbed by SRCR and SR-A (antigen adsorbed) (all *P *< 0.050) (Figure 3A, B). The decrease in the number of macrophages ingesting apoptotic cells correlated with the concentration of IgG that were positive for both anti-SRCR and anti-SR-A (*P *< 0.050), as shown in Figure 3C.

### Association of anti-class A scavenger receptors autoantibodies with clinical features of SLE

To investigate the associations between anti-class A scavenger receptors autoantibody and clinical manifestations or autoantibody profiles, those clinical features were compared between SLE patients positive and negative for anti-class A scavenger receptors autoantibodies. The clinical parameters refer to those present at the time of visit or during the 10 days before presentation, as defined by the SLEDAI-2K criteria. We identified no significant difference between SLE patients with and those without anti-class A scavenger receptors antibodies (anti-SRCR or anti-SR-A) in the presence of arthritis, serositis, mucosal ulcer, neurological or hematological manifestations, lupus nephritis, and lower levels of C3 or C4 (all *P *> 0.05). There was no association between anti-class A scavenger receptor autoantibody (anti-SRCR or anti-SR-A) and any of the autoantibodies such as anti-double-stranded DNA, anti-Sm, anti-RNP, anti-SSA, anti-SSB, anti-nucleosome, or anti-histone antibodies (all *P *> 0.05), either by a qualitative or a quantitative analysis.

## Discussion

Increased apoptosis and impaired phagocytic clearance of apoptotic cells could play important roles in the breakdown of self-tolerance in autoimmune diseases. Phagocytosis of apoptotic cells by macrophages is a tightly regulated process, which involves a range of receptors, ligands, and opsonins. Interestingly, some of the ligands and opsonins that are involved in the interaction between macrophages and apoptotic cells have been reported to be autoantigens in SLE patients. Reefman *et al. *[17] demonstrated that autoantibodies recognize antigens expressed on apoptotic cells and inhibit their uptake via an Fcγ receptor-dependent mechanism. Further, autoantibodies to serum components acting as bridging molecules (for example, C1q, mannose-binding lectin, serum amyloid P component, and C-reactive protein) have been detected in patients with SLE [18,19]. All these findings are indicative of the inhibitory effect of autoantibodies on the uptake of apoptotic cells by macrophages.

MARCO and SR-A are important binding receptors for apoptotic cells and contribute to their clearance by macrophages in the splenic marginal zone, an area where blood-borne apoptotic cells are trapped. Miyake *et al. *[20] found that the specifically ablation of the macrophages in the marginal zone delayed clearance of circulating apoptotic cell and impaired immune suppression to apoptotic cell antigens. With regard to that marginal zone macrophages characterized by high expression of MARCO and SR-A were responsible for apoptotic cell binding [21], Wermeling and colleagues [8] demonstrated that defects of MARCO and SR-A on these macrophages resulted in antinuclear autoimmunity. Moreover, Nicola and coworkers [22] observed that the defective expression of MARCO was linked to disease development via failure of apoptotic cell clearance in the splenic marginal zone in the SLE-prone mouse strain BXSB. These studies indicted that class A scavenger receptors expressed on the marginal zone macrophage play an important role in the link between apoptotic cells clearance and immune tolerance.

It is well known that autoantibodies have been found to precede the symptom onset of autoimmune diseases for many years in the case of SLE and RA patients [23,24]. Consistent with these observations, autoantibodies against scavenger receptors have been reported to emerge before the onset of clinical symptoms in SLE-prone mice [8]. Autoantibody against MARCO has been found in the sera of SLE patients [8]. It is intriguing to analyze the influences of anti-class A scavenger receptors antibodies on the clearance of apoptotic cells and its role in the pathogenesis of the disease.

In this study, autoantibodies to class A scavenger receptors, that is, anti-SRCR and anti-SR-A, can be found in patients with SLE (18.5% and 33.8%, respectively), RA (3.1% and 13.8%, respectively), and pSS (0% and 12.0%, respectively). These autoantibodies are more prevalent in patients with SLE. To explore the effects of anti-class A scavenger receptors antibodies on the clearance of apoptotic cells by macrophages, THP-1-derived macrophages that had been confirmed to express MARCO and SR-A were used to study the interaction between anti-class A scavenger receptors antibodies and macrophages. Our results indicated that phagocytic clearance of apoptotic cells by macrophages was inhibited by IgG positive for both anti-SRCR and anti-SR-A in a dose-dependent manner and partial removal of anti-SRCR and anti-SR-A antibodies decreased the inhibition effect of those IgG on phagocytosis; this suggests that the blockade of SR-A and the ligand-binding domain of MARCO by autoantibodies leads to the defective clearance of apoptotic cells. Treatment with anti-SRCR or anti-SR-A IgG led to significant inhibition effects on the binding of apoptotic cells by macrophages while only slightly effect on the ingestion, which may indicate that one of the class A scavenger receptors compensated for the other blocked one in the uptake of apoptotic cells. Though a wide range of receptors have been implicated in the uptake of apoptotic cell, including scavenger receptors of several classes, CD14, CD91, lectins, and the vitronectin receptor [25], expression of individual receptors can increase apoptotic cell adhesion with minimal effects on uptake [26]. It is possible that decreased binding of apoptotic cells due to impaired MARCO or SR-A would not reduce the uptake if one of the class A scavenger receptor is qualified. That can explain this discrepancy in the effect of autoantibodies on the binding and ingestion of apoptotic cells by macrophages. How these two receptors may interact to induce apoptotic cell ingestion needs further investigation.

Our study has also found that patients with pSS and RA have anti-class A scavenger receptors autoantibodies. This finding implies that these antibodies might play a role in the pathogenesis of autoimmunity. Anti-SR-A and anti-SRCR antibodies were more prevalent in patients with SLE, which is a disease characteristic of mouse models of impaired phagocytosis [27]. This suggests that defective phagocytic clearance of apoptotic cells by macrophages may be more remarkable and critical to triggering a pathologic autoimmune response in SLE than other autoimmune disease such as pSS or RA.

## Conclusions

This study demonstrates that class A scavenger receptors represent new autoantigens in SLE and other systemic autoimmune disorders. Autoantibodies to class A scavenger receptors might contribute to the breakdown of self-tolerance by impairing the clearance of apoptotic debris and play a role in the pathogenesis of autoimmune diseases, especially SLE. However, only an indirect linkage between these autoantibodies and the breakdown of self-tolerance has been established, and the function of these antibodies *in vivo *remains unclear. Additional studies are required to elucidate the complex interaction between anti-class A scavenger receptor antibodies and macrophage function in animal models.

## Abbreviations

7-AAD: 7-Amino-actinomycin D; ACR: American College of Rheumatology; CFSE: carboxyfluorescein diacetate succinimidyl ester; ELISA: enzyme-linked immunosorbent assay; FCS: fetal calf serum; MARCO: macrophage receptor with collagenous structure; OD: optical density; PBS: phosphate-buffered saline; PMA: phorbol 12-myristate 13-acetate; pSS: primary Sjögren syndrome; RA: rheumatoid arthritis; SD: standard deviation; SEM: standard error of mean; SLE: systemic lupus erythematosus; SLEDAI-2K: SLE disease activity index 2000; SR-A: scavenger receptor A; SRCR: scavenger receptor cysteine-rich.

## Competing interests

The authors declare that they have no competing interests.

## Authors' contributions

X-WC and YS performed most of the experiments and prepared the manuscript. C-YS worked on the clinical data presentation and participated in the statistical analysis. C-DY was responsible for the main experimental design, data interpretation, and for finalizing the manuscript. All authors read and approved the final manuscript.

## Supplementary Material

Additional file 1 **Clinical and serological features of 65 SLE patients**. Main clinical and serological features of SLE patients have been shown in this additional file. Click here for file
