# Supplementary material for: Anti-class a scavenger receptor autoantibodies from systemic lupus erythematosus patients impair phagocytic clearance of apoptotic cells by macrophages in vitro
Source: Arthritis Res Ther. 2011 Jan 31;13(1):R9. doi: 10.1186/ar3230 (PMC3241353; doi:10.1186/ar3230)
Supplement: Additional file 1 — Clinical and serological features of 65 SLE patients. Main clinical and serological features of SLE patients have been shown in this additional file. [file ar3230-S1.DOC]

**Additional file 1**

|  | Numberof patients (%) |
| --- | --- |
| Arthritis | 22 (33.8) |
| Rash | 40 (61.5) |
| Serositis | 16 (24.6) |
| CNS involvement | 8 (12.3) |
| Renal involvement | 27 (41.5) |
| Hematologic manifestation | 52 (80.0) |
| Cutaneous vasculitis | 11 (16.9) |
| Mucosal ulcers | 5 (7.7) |
| Fever | 18 (27.7) |
| Low C3/C4 | 43 (66.2) |
| Anti-nuclear antibodies | 64 (98.5) |
| Anti-dsDNA antibodies | 62 (95.4) |
| Anti-Sm | 23 (35.4) |
| Anti-SSA | 19 (29.2) |
| Anti-SSB | 10 (15.4) |
| Anti-nucleosome | 21 (32.3) |
| Anti-histone | 23 (35.4) |
| Anti-RNP | 9 (13.8) |

Clinical and serological features of 65 SLE patients.

CNS involvement: cerebrovascular disease, seizure, psychosis or anxiety disorder.

Renal involvement: proteinuria (>500 mg/24 hour) or hematuria.

Hematologic manifestation: Leukopenia (<4,000 cells/mm^3^) or Thrombocytopenia (<100,000 cell/mm^3^).

The percentage refers to the presence of the manifestation at the time of visit or in the preceding 10 days.
